# Lignin-Assisted Dispersion and Iohexol Retention in Lead-Free Bi_2_O_3_/Iohexol-Loaded Waterborne Polyurethane Sheets for Diagnostic X-Ray Attenuation

**DOI:** 10.3390/polym18192359

**Published:** 2026-09-28

**Authors:** Seon-Chil Kim

**Affiliations:** Department of Biomedical Engineering, School of Medicine, Keimyung University, 1095 Dal-gubeol-daero, Daegu 42601, Republic of Korea; chil@kmu.ac.kr; Tel.: +82-10-4803-7773

**Keywords:** lead-free shielding, lignin, bismuth oxide, iodine contrast component, diagnostic X-ray

## Abstract

Conventional lead-based protective materials used in diagnostic radiology involve some notable limitations in terms of high weight, toxicity, and environmental concerns associated with their disposal. Lead-free polymer shielding materials incorporating high-atomic-number fillers and iodine-containing components have therefore attracted increasing attention. However, high filler loading can induce particle agglomeration, localized iodine-rich regions, interfacial defects, and nonuniform X-ray attenuation. Accordingly, improving filler dispersion and structural stability is essential to maintain effective shielding performance. In this study, the effects of lignin-assisted interfacial stabilization on filler dispersion, iodine retention, structural properties, and X-ray shielding performance in highly filled polyurethane (PU) composites were investigated. At 80 kVp, a 3 wt% lignin sheet exhibited a radiation protection efficiency of 77.4%, a linear attenuation coefficient of 48.52 cm^−1^, a half-value layer (HVL) of 0.143 mm, and a mean free path (MFP) of 0.206 mm. These results indicate that lignin functions as a bio-derived interfacial stabilizer rather than a primary X-ray attenuator, improving filler utilization through enhanced dispersion, packing structure, and iodine immobilization. The proposed lignin-assisted PU/Bi_2_O_3_/iodine composite therefore shows potential as an environmentally conscious auxiliary shielding material for diagnostic X-rays and low-dose scattered radiation.

## 1. Introduction

Diagnostic X-rays are widely used across various medical imaging applications such as general radiography, interventional procedures, and computed tomography (CT). They play an essential role in the early diagnosis of diseases and clinical decision-making during treatment [1]. However, given that X-rays are a form of ionizing radiation, unnecessary or repeated exposure may pose biological risks to both patients and healthcare workers [2]. Therefore, the use of medical radiation requires adherence to radiation protection principles designed to maintain diagnostic benefits while minimizing radiation exposure [3]. The energy range of diagnostic X-rays varies depending on the imaging modality and anatomical region being examined. Diagnostic imaging is typically performed with low- to intermediate-energy X-rays that generally range from approximately 20 to 125 keV [4]. Within this energy range, X-ray attenuation is influenced by several material and structural parameters including the atomic number, density, thickness, and electron density of the shielding material as well as filler dispersion and interfacial stability between the fillers and polymer matrix [5]. Accordingly, more effective absorption and scattering of incident X-rays within a material improve its performance in terms of radiation shielding by reducing the dose transmitted through the material [6].

Radiation protection devices currently used in clinical settings have traditionally relied on lead-based materials. Some common configurations include protective garments, thyroid shields, gloves, and gonadal shields [7]. Although lead provides excellent X-ray attenuation because of its high atomic number and density, it is associated with several drawbacks such as toxicity, high weight, hardening and cracking under repeated flexural stress, discomfort on the part of wearers, and environmental concerns related to end-of-life disposal [8]. These limitations have motivated further research on lead-free X-ray shielding materials [9]. In particular, polymer composites are considered promising alternatives for diagnostic X-ray shielding because of their processability, flexibility, lightweight characteristics, and tunable filler compositions [10]. Polymer-based shielding composites also offer some notable advantages in terms of the capability to tailor attenuation characteristics to specific diagnostic X-ray energy ranges by controlling parameters such as filler type and loading, particle size, sheet thickness, multilayer structure, and interfacial stabilization strategies [11].

High-atomic-number inorganic fillers including tungsten, bismuth, barium, antimony, and gadolinium have been extensively investigated as alternatives to lead for radiation shielding in practical applications [12]. These high-Z fillers can enhance shielding performance by increasing the probability of interactions with X-rays. However, when incorporated at high loadings into a polymer matrix, they can induce particle agglomeration, polymer–particle interfacial debonding, microvoid formation, localized low-density regions, and reduced sheet flexibility [13,14]. In particular, nonuniform filler dispersion can result in excessive concentrations of shielding components in some regions while creating poorly shielded regions with insufficient filler content in others. These low-shielding regions may provide preferential pathways for X-ray transmission, which can result in actual shielding performance falling below theoretical expectations despite the presence of high-Z particles. Therefore, the development of advanced polymer-based shielding materials requires the development of effective interfacial strategies designed to enable their uniform dispersion and long-term stabilization within the polymer matrix in addition to increasing the loading of high-Z fillers.

In this study, we designed a polyurethane-based lead-free shielding sheet incorporating iodine-containing components, bismuth oxide, and lignin to address these challenges. Iodine was selected as the first functional component based on its atomic number of 53 and long history of widespread use as a major constituent of X-ray and CT contrast agents in medical imaging [15]. In particular, iodine exhibits a K-edge within the diagnostic X-ray energy range that enables it to contribute effectively to X-ray attenuation over specific energy ranges [16]. Previous studies have reported that incorporating iodine-based contrast media into polymer matrices increases X-ray absorption and the linear attenuation coefficient with increasing iodine content [17]. However, introducing high concentrations of iodine-containing components into a polymer matrix may compromise the internal homogeneity of the sheet and promote the formation of iodine-rich domains or iodine agglomerates [18]. Therefore, to improve the effective shielding performance of iodine-based shielding sheets, new strategies must be developed to achieve uniform iodine distribution and stable immobilization within the polymer matrix.

Bismuth oxide (Bi_2_O_3_) was selected as the second functional component as a high-Z lead-free shielding filler. Bi_2_O_3_ is well suited for attenuating diagnostic X-rays and is considered a promising lead-free alternative that can reduce the toxicity and environmental burden associated with conventional lead-based shielding materials owing to its high atomic number and relatively high density of approximately 8.90 g/cm^3^ [19]. In addition, Bi_2_O_3_ can be dispersed and immobilized in powder form within polymer matrices, which makes it suitable for the fabrication of flexible shielding sheets [20]. However, because Bi_2_O_3_ is a high-density inorganic particulate filler, incorporation at high loadings may lead to particle agglomeration, sedimentation within the polymer matrix, interfacial defects, and reduced sheet flexibility [21]. Therefore, the effective use of Bi_2_O_3_ requires adequate interfacial stability with the polymer matrix together with an appropriate additive strategy to promote its uniform distribution with other functional shielding components.

Lignin was selected as the third functional component. This material is a natural aromatic polymer derived from lignocellulosic biomass [22]. Lignin contains phenolic hydroxyl groups, aliphatic hydroxyl groups, and aromatic rings, which can facilitate hydrogen bonding, π-interactions, and other interfacial interactions with polymer matrices and functional fillers [23]. In this study, lignin was not intended to serve as a primary shielding component for direct X-ray attenuation. Rather, it was designed as a biologically derived interfacial stabilizer to improve the dispersion of the iodine-containing component and Bi_2_O_3_ particles and to reduce microvoids, filler-rich agglomerates, and poorly shielded regions within the polyurethane matrix. In particular, the aromatic structure and hydroxyl groups of lignin may interact with the iodine-containing component and the polyurethane (PU) matrix. This can help mitigate the formation of iodine-rich agglomerates and potentially reduce the mobility and leaching of the iodine-containing component [24].

PU was used as the polymer matrix in this study. PU is well suited for use as a matrix for wearable or in attachable medical radiation shielding materials owing to its flexibility, elasticity, coating capability, and favorable processability into sheet structures [25]. In particular, PU-based sheets can maintain flexible shielding structures even at high inorganic filler loadings through processing techniques such as coating, casting, hot pressing, and the formation of protective surface layers [26]. In the present study, Bi_2_O_3_ was employed as the primary shielding filler with the iodine-containing component as an auxiliary attenuating component for low-energy X-rays and lignin as a bio-derived dispersion stabilizer and interfacial modifier. Through this material design, we aimed to establish a lignin-assisted interfacially stabilized structure rather than a simple physical mixture of high-Z fillers and iodine-containing components. We adopted this approach to improve X-ray shielding performance and structural homogeneity simultaneously.

Therefore, in this study, we varied the lignin content of the experimental protective shielding from 0 to 5 wt% in PU-based lead-free shielding sheets containing fixed amounts of Bi_2_O_3_ (50 wt%) and an iodine-containing component (30 wt%) to evaluate the effects of lignin on the dispersion, interfacial stability, iodine retention, and low-energy diagnostic X-ray shielding performance of the highly loaded iodine/Bi_2_O_3_ filler. Previous studies have focused primarily on increasing iodine content, synthesizing iodinated polymers, or incorporating high-atomic-number inorganic fillers. In this work, we propose an interfacial design strategy using bio-derived lignin to reduce filler agglomeration and poorly shielded regions in highly filled shielding composites to improve their effective shielding performance. This approach provides a new design strategy for flexible, lead-free auxiliary shielding sheets applicable to low-energy diagnostic X-ray and low-dose scattered radiation environments.

This study considered three primary objectives. First, we evaluated the low-energy diagnostic X-ray attenuation performance of PU shielding sheets containing Bi_2_O_3_ and an iodine-containing component. Second, we also investigated the effects of lignin content on the dispersion of the iodine/Bi_2_O_3_ fillers, interfacial stability, and internal homogeneity of the sheets. Finally, we comprehensively assessed X-ray transmittance, radiation protection efficiency (RPE), linear attenuation coefficient, mass attenuation coefficient, half-value layer (HVL), mean free path (MFP), mass per unit area, and iodine leaching stability under diagnostic X-ray conditions ranging from 40 to 120 kVp. The results of these investigations demonstrate the potential of an environmentally conscious, flexible, lead-free auxiliary shielding sheet for low-dose medical radiation and low-energy scattered X-ray environments. In particular, the findings establish the effectiveness of the lignin-assisted interfacial stabilizer in enhancing dispersion stability and effective X-ray shielding performance.

## 2. Materials and Methods

Polyurethane (PU) was used as the flexible polymer matrix, while bismuth oxide (Bi_2_O_3_), an iodine-containing contrast agent, and lignin were incorporated as functional components of the shielding composite. A waterborne polyurethane dispersion or an equivalent coating-grade PU was used as the polymer matrix, and all compositions were calculated in weight percent (wt%) based on the dry solids content of the shielding core layer.

The iodine-containing component was not crystalline elemental iodine (I_2_), but rather an iodinated contrast agent in which iodine atoms are covalently bound within an organic molecular structure. In this study, iohexol powder was used as the iodine-containing component, and the specified loading of 30 wt% was defined based on the dry mass of iohexol powder incorporated into the shielding core layer. Bi_2_O_3_ was used as the primary shielding filler owing to its qualities as a lead-free high-Z material with a high atomic number and density. This material was included to contribute directly to the attenuation of the diagnostic X-ray energy [27]. The iodine-containing component was incorporated as an auxiliary attenuating component, particularly for the low-energy X-ray range. Organosolv lignin (CAS No. 8068-03-9; Leapchem, Hangzhou, China) was supplied in powder form. This component is an environmentally friendly lignin isolated from plant biomass using an organic solvent-based organosolv process. The lignin was incorporated as a bio-derived auxiliary material to improve filler dispersion, reduce microvoid formation, and enhance iodine retention stability within the PU matrix containing Bi_2_O_3_/iohexol fillers. However, the independent contribution of the lignin to X-ray attenuation could not be quantified directly because we did not test a separate control group containing lignin alone in the PU matrix in this study.

A key feature of the experimental design was that the Bi_2_O_3_ and iodine-containing component loadings were fixed at 50 wt% and 30 wt%, respectively, while only the lignin content was varied. This design enabled us to determine whether changes in shielding performance were attributable to lignin-induced improvements in particle dispersion, stability, and structural homogeneity rather than simply to increased Bi_2_O_3_ or iodine-containing component loading. The compositions of the shielding core layers were designed as summarized in Table 1.

Accordingly, the fabricated specimens L0, L1, L2, L3, and L5 constituted the primary experimental groups. Where feasible, PU-Bi50 and PU-I30 specimens were also prepared and used as control groups to evaluate the contributions of Bi_2_O_3_ and the iodine component to the overall X-ray attenuation performance separately. The compositions of the specimens were calculated on a dry-solid basis. For example, assuming that the waterborne polyurethane (WPU) dispersion contained 35 wt% solids, preparation of the L2 specimen required 5.00 g of Bi_2_O_3_, 3.00 g of the iodine component, 0.20 g of lignin, and 5.14 g of WPU dispersion to provide 1.80 g of PU solids. In this formulation, lignin was employed as a dispersion aid for the iodine component rather than merely as an additive. Therefore, a lignin–iodine pre-dispersion was prepared prior to incorporation into the composite. Lignin was first dispersed in an ethanol/water mixed solvent with respective volume fractions of 60vol% and 40vol%, after which iohexol powder was gradually added to the lignin dispersion. The resulting mixture was stirred for approximately 60min followed by an additional 15 min of ultrasonic dispersion.

This process was designed based on the potential of the aromatic structure and hydroxyl groups of lignin to contribute to non-covalent interfacial interactions with the iodine-containing component, Bi_2_O_3_, and the PU matrix. However, because the present study did not directly demonstrate interactions between lignin and iohexol or between lignin and Bi_2_O_3_, the role of lignin was interpreted only indirectly based on the combined results of FESEM–EDS mapping, ImageJ-based analysis of distribution uniformity, reduced iodine leaching, and improved X-ray shielding performance [28].

The lignin content was varied at 0, 1, 2, 3, and 5 wt%. The L1 specimen was designed to evaluate the effect of low lignin loading, L2 and L3 were selected to investigate the potential optimal lignin-loading range, and L5 was included to assess possible limitations associated with excessive lignin addition such as increased agglomeration and brittleness.

To fabricate the final shielding sheets, Bi_2_O_3_ powder was gradually added to the lignin–iodine pre-dispersion followed by high-speed stirring for at least 30 min to achieve primary dispersion. Secondary dispersion was then performed for 15 min using either an ultrasonic bath or a probe sonicator. The WPU solution was subsequently added, and the mixture was stirred for 60 min. After mixing, entrapped air bubbles were removed by vacuum degassing or low-speed centrifugal degassing. The resulting mixture was cast onto a release film using a doctor blade to obtain a uniform thickness and then dried in a vacuum oven at 40 °C for 12 h. After drying, the sheet was peeled from the release film and hot-pressed at 50 °C to improve thickness uniformity.

Finally, a thin PU top coating was applied to both surfaces of the sheet to minimize iodine leaching, surface contamination, and filler loss. The compositions presented in Table 1 refer exclusively to the shielding core layer, whereas the PU top coating was considered a separate protective layer and was not included in the core-layer composition. The final shielding sheets were fabricated with dimensions of 300 mm × 300 mm × 0.3 mm (Figure 1).

The fabricated shielding sheets were cut into specimens of identical area, and their thickness, mass, mass per unit area, and apparent density were measured. The mass per unit area was calculated according to Equation (1) [29].

(1) $$Areal\;density=\frac{m}{A},$$

where *m* is the mass of the specimen and *A* is the area of the specimen. The apparent density (ρ) was calculated using Equation (2) [30].

(2) $$\rho =\frac{m}{A\times x}$$

where x is the average thickness of the specimen. When evaluating the effect of lignin, the analysis was not limited to changes in apparent density; shielding performance was also compared under conditions of equivalent thickness and equivalent mass per unit area.

Field-emission scanning electron microscopy (FESEM) was used to investigate the dispersion of Bi_2_O_3_, the iodine-containing component, and lignin within the fabricated sheets [31]. The specimens were cryogenically fractured in liquid nitrogen to expose their cross-sectional surfaces and were then coated with carbon or another thin conductive layer to ensure electrical conductivity for analysis with energy-dispersive X-ray spectroscopy (EDS). The surface and cross-sectional morphologies of the sheets were subsequently examined using an FESEM instrument (Hitachi S-4800, Hitachi, Tokyo, Japan). The distributions of Bi_2_O_3_ and the iodine-containing component were analyzed using EDS elemental mapping. The Bi signal was used as an indicator of Bi_2_O_3_ distribution, whereas the I signal was used to assess the distribution of the iodine-containing component. In particular, PU-Bi-I-L0 was compared with PU-Bi-I-L2 and PU-Bi-I-L3 to evaluate whether the addition of lignin reduced iodine-rich agglomeration and Bi_2_O_3_ particle aggregation.

The EDS mapping images were quantitatively analyzed using ImageJ (version 1.54). For images acquired at the same magnification for each specimen, regions of interest (ROI) of identical size were defined after excluding the scale bar and sample labels. The mean intensities and coefficients of variation (CV) of the Bi and I signals were calculated. A lower CV value was considered indicative of a more homogeneous elemental distribution. In addition, the Bi/I-rich agglomerate area fraction and the low-shielding region fraction defined as areas exhibiting simultaneously low Bi and I signals were quantified to evaluate the formation of poorly shielded regions within the shielding layer.

The mechanical properties of the shielding sheets were evaluated in accordance with ASTM D882 [32]. ASTM D882 is a standard testing method to determine the tensile properties of thin plastic sheeting and films and was considered appropriate for the approximately 0.3-mm-thick PU composite sheets used in this study. Tensile testing was performed using a universal testing machine (AGX-V, Shimadzu Corp., Kyoto, Japan, 2019). Strip specimens of uniform width and length were cut from the fabricated 300 mm × 300 mm sheets, and the crosshead speed was set to 200 ± 10 mm/min. At least three specimens were tested for each composition, and the tensile strength, Young’s modulus, and elongation at break were expressed as the mean ± standard deviation.

A repeated flexural durability test was performed to evaluate the suitability of the shielding sheets for wearable or attachable applications. The test was conducted as a modified cyclic flexing test based on the bend-flexing concept of ASTM D1052 using a universal testing machine equipped with a cyclic flexing jig [33,34]. The specimens were secured using upper and lower circular clamps or a flexing jig and subjected to 5000 flexing cycles under the specified displacement conditions. Changes in the surfaces of the specimens before and after cyclic flexing were examined, and the retention of X-ray shielding performance was calculated according to Equation (3) based on the RPE measured at 80 kVp. An appropriate lignin content was expected to improve mechanical durability by enhancing interfacial stability, whereas excessive addition of lignin could increase the brittleness of the sheet or lead to more agglomeration-related defects [35]. Therefore, the L5 specimen was included as a comparison group to evaluate the potential limitations associated with excessive lignin loading (Figure 2).

(3) $${RPE}_{reten}(\%)=\frac{{RPE}_{after\;flex}}{{RPE}_{bcfore\;flex}}\times 100$$

An iodine leaching test was performed to evaluate the practical applicability of the iodine-containing shielding sheets. Specimens of uniform dimensions (10 mm × 10 mm × 0.3 mm) were immersed in distilled water and maintained at 37 °C for respective durations of 24 h, 72 h, 7 days, and 14 days. The concentration of iodine in the immersion solution was analyzed using inductively coupled plasma optical emission spectrometry (ICP-OES), and the X-ray shielding performance of the specimens was also compared after immersion. A reduction in iodine leaching and greater retention of X-ray shielding performance after immersion in the lignin-containing groups would indicate the stabilizing effect of lignin on the iodine-containing component.

Cumulative iodine leaching was calculated according to Equation (4).

(4) $$Iodine\;Leaching(\%)=\frac{M_{I\cdot leached}}{M_{I\cdot initial}}\times 100.$$

$M_{I\cdot leached}$ represents the cumulative amount of iodine leached into the immersion solution, and $M_{I\cdot initial}$ represents the initial iodine content in the specimen.

The experimental setup used to evaluate radiation shielding performance was configured as shown in Figure 3 in accordance with the Korean Industrial Standard KS A 4025:1990 (confirmed in 2009) testing method for lead-equivalent X-ray protective devices [36]. An X-ray generator (Toshiba E7239, 150 kV–500 mA, Toshiba, Kawasaki, Japan, 1999) was used, and measurements were repeated at least three times under each experimental condition with the mean values used for the analysis. A calibrated dose detector (DosiMax plus 1, IBA Dosimetry Corp., Schwarzenbruck, Germany, 2019) was used to measure the radiation dose.

The initial radiation dose $I_{0}$ was measured without a specimen, and the transmitted radiation dose *I* was measured after placing the specimen in front of the detector. For each experimental condition, the transmittance, RPE, linear attenuation coefficient, mass attenuation coefficient, HVL, and MFP were calculated and quantitatively compared [37,38].

(5) $$T=\frac{I}{I_{0}},$$

(6) $$RPE(\%)=(1-\frac{I}{I_{0}})\times 100,$$

(7) $$\mu =-\frac{1}{\chi }ln(\frac{I}{I_{0}}),$$

(8) $$\mu_{m}=\frac{\mu }{\rho },$$

(9) $$HVL=\frac{0.693}{\mu },$$

(10) $$MFP=\frac{1}{\mu }.$$

Here, T represents the transmittance (Equation (5)), RPE represents the radiation protection efficiency (Equation (6)), μ is the linear attenuation coefficient (Equation (7)), χ is the sheet thickness, ρ is the apparent density, and $\mu_{m}$ is the mass attenuation coefficient (Equation (8)). Accordingly, shielding performance was evaluated by comparisons at equivalent thickness, at equivalent mass per unit area, and between sheets with and without lignin at the same Bi_2_O_3_ and iodine-containing component loadings.

In addition to RPE, the HVL and MFP were calculated to quantitatively compare the effective attenuation performance of the shielding sheets. The HVL (Equation (9)) represents the thickness of shielding material required to reduce the initial X-ray intensity by half, and a lower HVL indicates that the same attenuation effect can be achieved with a thinner shielding layer [39]. The MFP (Equation (10)) represents the average distance traveled by an X-ray photon within a material before undergoing an interaction, and a lower MFP indicates that photons are absorbed or scattered over a shorter distance within the composite sheet [40]. Therefore, reductions in HVL and MFP following lignin addition were used as indicators to evaluate whether lignin improved the dispersion and stability of the iodine-containing component and Bi_2_O_3_ fillers.

## 3. Results

The macroscopic appearance of the fabricated sheets showed no visually distinguishable differences associated with lignin content, and all specimens maintained a thin, film-like structure with a thickness of approximately 0.3 mm. The morphology and distribution of the shielding components within the sheets were further examined by FESEM analysis of the surface and cross-sectional regions to assess the dispersion of Bi_2_O_3_ and the iodine-containing component. As shown in Figure 4, no severe surface cracking or delamination was observed in the sheets containing 1–3 wt% lignin. However, in the sheet containing 5 wt% lignin, although only minor surface irregularities were visible after casting, localized heterogeneity including small agglomerated regions was observed within the sheet as shown in Figure 4E(a). These findings suggest that excessive lignin addition may reduce dispersion stability within the PU matrix.

As shown in Figure 5, the lignin-free PU-Bi-I-L0 specimen frequently exhibited localized Bi-rich regions with bright contrast and iodine-rich domains as indicated in Figure 5A(a). Such localized agglomeration of high-Z components may produce strong attenuation in certain regions while creating poorly shielded pathways with relatively low filler concentrations in other regions. In contrast, PU-Bi-I-L2 and PU-Bi-I-L3 exhibited relatively more uniform distributions of Bi and I in the EDS elemental mapping together with reductions in large agglomerates and microvoids. These findings suggest that the addition of lignin may have improved the uniformity of the dispersion of the iodine-containing component and Bi_2_O_3_ particles as well as their packing structure within the PU matrix. However, these findings should be regarded as an indirect interpretation based on the observed morphological characteristics and elemental distribution because the interactions between lignin and the iodine-containing component or between lignin and Bi_2_O_3_ were not directly verified by spectroscopic analyses.

The uniformity of the elemental distributions of Bi and I was evaluated using the CV by dividing each EDS map into grids of identical size and calculating the mean elemental intensity within each grid. A lower CV indicates a more uniform elemental distribution. As shown in Table 2, the CV of the iodine distribution was 18.6% for L0 but decreased to 9.8% and 8.5% for L2 and L3, respectively. Similarly, the CV of the Bi distribution decreased from 15.4% for L0 to approximately 8% for both L2 and L3. These results indicate that a lignin content of 2–3 wt% may represent the most effective range to improve the dispersion uniformity of the iodine/Bi_2_O_3_ hybrid fillers.

The void area fraction was 3.7% for L0 but decreased to 1.6% and 1.4% for L2 and L3, respectively. This finding suggests that lignin may enhance the stability of the filler–PU interface and thereby reduce void formation and microstructural defects. However, both the CV and void area fraction increased again for L5, which suggests that excessive lignin addition may compromise structural homogeneity, possibly because of lignin self-agglomeration or a reduction in the relative PU matrix content.

As shown in Table 3, the physical properties of the fabricated sheets did not differ substantially in overall terms, although quantitative differences were observed among the compositions. The apparent density of PU-Bi-I-L0 was 2.52 g/cm^3^, whereas it increased to 2.69 and 2.73 g/cm^3^ with the addition of 2 and 3 wt% lignin, respectively. This increase may be attributed to improved dispersion stability of Bi_2_O_3_ and the iodine-containing component and a reduction in microvoids within the PU matrix that resulted in increased packing density. In contrast, the apparent density of PU-Bi-I-L5 decreased to 2.62 g/cm^3^, which suggests that excessive lignin addition may reduce the densification effect because of increased agglomeration or void formation. These results are consistent with previous studies on polymer-based radiation shielding composites. This indicates that shielding performance is determined not only by the loading of high-density fillers but also by the uniformity of filler dispersion within the polymer matrix [41].

Furthermore, because the shielding sheets are intended for use as wearable or attachable medical radiation protection materials, their tensile strength and retention of shielding performance after repeated flexing were evaluated in addition to their shielding performance [42]. The mechanical properties of the fabricated shielding sheets are presented in Table 4.

Given that the PU-Bi-I-L0 contained a total of 80 wt% Bi_2_O_3_ and the iodine-containing component, its tensile strength and elongation at break were substantially lower than those of PU-I30. This reduction may be attributed to the high loading of inorganic and iodine-containing fillers, which can disrupt the continuity of the PU matrix and increase interfacial defects. However, with the addition of 2 and 3 wt% lignin, the tensile strength respectively increased to 10.9 and 11.2 MPa, and the retention of RPE after 5000 flexing cycles improved to more than 97%. These results suggest that lignin may contribute to interfacial stabilization between the PU matrix and the fillers rather than acting merely as a filler and thereby reduce filler loss, microcrack formation, and localized shielding defects that may occur during repeated flexing [43]. In contrast, the L5 specimen exhibited decreases in both tensile strength and elongation at break together with increased mass loss after flexing. This behavior may be attributed to excessive lignin forming agglomerates or brittle regions within the PU matrix, which would have reduced the mechanical stability.

Iodine leaching was assessed under distilled-water immersion conditions to evaluate the practical applicability of the iodine-containing shielding sheets, and the results are presented in Table 5.

In the lignin-free L0 specimen, cumulative iodine leaching reached 3.8% after 14 days, whereas it decreased to 1.3% and 1.1% in L2 and L3, respectively. This reduction may be attributed to interactions between the aromatic structure and hydroxyl groups of lignin and the iodine-containing component, which may reduce the mobility of the iodine-containing component within the PU matrix.

In particular, the L3 specimen exhibited the lowest iodine leaching and the highest retention of shielding performance after immersion. However, the leaching rate increased again in L5, which suggests that excessive lignin addition may increase matrix heterogeneity and microvoid formation and thereby create pathways for water penetration.

For the evaluation of X-ray shielding performance, the initial radiation dose $I_{0}$ was measured without a specimen, and the transmitted radiation dose I was measured after placing the specimen in front of the detector. RPE was then calculated as presented in Table 6.

The RPE of the shielding sheets decreased with increasing tube voltage. This trend can be attributed to the increased penetrating ability of X-ray photons with increasing photon energy and the corresponding decrease in the relative contribution of the photoelectric effect, which predominates in the low-energy X-ray region.

PU-I30 exhibited RPE values of 42.6% at 40 kVp and 28.4% at 80 kVp. These results indicate that the iodine-containing component alone did not suffice to provide adequate shielding performance in a 0.3-mm-thick sheet. In contrast, PU-Bi50 exhibited an RPE of 55.6% at 80 kVp, which confirms that Bi_2_O_3_ served as the primary shielding filler. PU-Bi-I-L0, which contained both Bi_2_O_3_ and the iodine-containing component, exhibited an RPE of 66.8% at 80 kVp and thus demonstrated higher shielding performance than PU-Bi50 and PU-I30. This improvement may be attributed to the combined effects of the high atomic number and high density of Bi_2_O_3_ and the X-ray attenuation characteristics of the iodine-containing component in the low-energy region.

A comparison of the PU-Bi50 and PU-I30 control groups showed that PU-Bi50 exhibited a higher RPE than PU-I30 at all tube voltages, which indicates that Bi_2_O_3_ served as the primary shielding filler in the composite sheets. However, PU-Bi-I-L0, which contained both Bi_2_O_3_ and iohexol, exhibited a higher RPE than PU-Bi50. This confirms that the addition of iohexol contributed to further X-ray attenuation. These findings suggest that Bi_2_O_3_ provided the primary attenuation effect, while iohexol contributed additional attenuation in the low-energy diagnostic X-ray range. Furthermore, the improved effective shielding performance observed with lignin addition may be attributed to enhanced dispersion stability of the two fillers.

The lignin-containing PU-Bi-I-L2 and PU-Bi-I-L3 specimens exhibited higher RPE values than L0 at all tube voltages. In particular, at 80 kVp, the RPE increased from 66.8% for L0 to 75.6% and 77.4% for L2 and L3, respectively. These results demonstrate that lignin addition can improve shielding performance even at the same Bi_2_O_3_ and iodine-containing component loadings of 50 wt% and 30 wt%, respectively. This improvement does not imply that lignin itself acts as a high-Z shielding material. Rather, it suggests that lignin improves the dispersion of the iodine-containing component and Bi_2_O_3_ fillers thereby reduces regions with relatively high X-ray transmission within the shielding sheet.

The quantitative shielding parameters of the specimens at this tube voltage were compared as shown in Table 7 because a value of 80 kVp was considered as a relevant condition to evaluate shielding against diagnostic X-rays and scattered radiation.

At 80 kVp, PU-Bi-I-L3 exhibited the lowest transmittance, the highest linear attenuation coefficient, and the lowest HVL and MFP values. Compared to L0, the RPE of L3 increased from 66.8% to 77.4%, while the linear attenuation coefficient increased from 36.36 cm^−1^ to 48.52 cm^−1^. In addition, the HVL decreased from 0.191 mm to 0.143 mm. These results indicate that the L3 specimen attenuated X-rays more effectively than L0 with a sheet of the same thickness.

As shown in Figure 6, L2 exhibited shielding performance comparable to that of L3, with an RPE of 75.6% and an HVL of 0.150 mm. Given the relatively small difference in shielding performance between L2 and L3, the optimal composition should be determined by considering not only shielding performance but also mechanical flexibility, manufacturing stability, iodine leaching stability, and cost. In contrast, L5 exhibited a lower μ and a higher HVL than L2 and L3. These results suggest that lignin contributes to interfacial stabilization and improved filler dispersion up to an appropriate loading level, whereas excessive lignin addition may reduce shielding performance because of a decreased proportion of the PU matrix, lignin self-agglomeration, increased void formation, or less efficient filler packing.

## 4. Discussion

In this study, we achieved improved effective shielding performance by using lignin to control the dispersion and stability of the iodine/Bi_2_O_3_ hybrid fillers. Accordingly, lignin was employed as a bio-derived interfacial stabilizer to facilitate dispersion stability, interfacial interactions, microvoid reduction, and suppression of iodine leaching in iodine- and Bi_2_O_3_-containing PU shielding sheets rather than as a primary component for direct X-ray attenuation. The aromatic structure and hydroxyl groups of lignin provide the potential for interactions with the iodine-containing component, Bi_2_O_3_ particles, and the PU matrix, which can potentially contribute to reductions in filler-rich agglomerates and low-shielding regions in composite sheets containing high loadings of shielding fillers [44].

In general, the shielding performance of polymer-based X-ray shielding composites increases with increasing loadings of fillers having high atomic numbers and high densities [45]. However, high filler loadings can promote agglomeration, sedimentation, interfacial debonding, microvoid formation, and the development of low-shielding regions within the polymer matrix, which may increase the actual transmitted radiation dose [46]. Therefore, in this study, the Bi_2_O_3_ and iodine-containing component loadings were fixed at 50 wt% and 30 wt%, respectively, to maintain sufficiently high loadings of high-atomic-number shielding components, while only the lignin content was varied from 0 to 5 wt% to independently evaluate the auxiliary effect of lignin. This experimental design was intended to determine how effectively lignin enables the shielding fillers to function uniformly at identical Bi_2_O_3_ and iodine-containing component loadings rather than to examine whether lignin acts as a primary shielding component.

FESEM- energy dispersive X-ray spectroscopy (EDS) analysis showed that the lignin-free specimen exhibited localized Bi-rich regions and iodine-rich domains, which suggests the potential formation of low-shielding pathways. In contrast, the specimens containing 2–3 wt% lignin exhibited reductions in the CV of the I and Bi distributions, void area fraction, and agglomerate area fraction. These findings indicate that lignin acted as a stabilizing additive that improved the dispersion of the iodine-containing component and Bi_2_O_3_ particles while reducing microvoids and agglomeration-related defects within the PU matrix. These microstructural improvements were also reflected in the physical properties and mechanical durability of the composite sheets. The specimens containing 2–3 wt% lignin exhibited higher apparent densities than the lignin-free specimen, which may be attributed to improved filler packing and reduced void formation rather than simply an increase in mass. In addition, the tensile strength and retention of shielding performance after repeated flexing were improved. This suggests that lignin can enhance the dispersion stability and mechanical durability of the PU matrix, which may otherwise be compromised by the high loadings of Bi_2_O_3_ and the iodine-containing filler. In particular, because wearable or attachable shielding sheets may be subjected to repeated flexing and deformation during use, the high retention of RPE after flexing is particularly relevant to their potential practical applicability [47].

Iodine leaching stability is another important finding of this study. Although iodine-containing shielding sheets are advantageous for low-energy X-ray attenuation, the iodine-containing component may leach when exposed to external aqueous environments during use if it is not sufficiently stabilized within the polymer matrix [48]. In this study, the specimens containing 2–3 wt% lignin exhibited substantially lower iodine leaching after 14 days than the lignin-free specimen while maintaining higher RPE retention after immersion. These findings may be attributed to interactions between the aromatic structure and hydroxyl groups of lignin and the iodine-containing component together with reductions in voids and agglomerated structures within the PU matrix, which may restrict the mobility of the iodine-containing component.

The evaluation of X-ray shielding performance demonstrated the combined effect of Bi_2_O_3_ and the iodine-containing component, together with a clear improvement in effective attenuation performance following lignin addition. At 80 kVp, PU-Bi-I-L0 exhibited an RPE of 66.8%, whereas the RPE increased to 75.6% and 77.4% with the addition of 2 wt% and 3 wt% lignin, respectively. In addition, the L3 specimen exhibited an increased linear attenuation coefficient and decreased HVL and MFP values. This indicates that X-rays were attenuated more effectively over a shorter distance at the same sheet thickness.

Therefore, this study quantitatively evaluated the role of lignin under fixed Bi_2_O_3_ and iodine-containing component loadings. The L2 and L3 specimens showed favorable results in terms of EDS distribution uniformity, void area fraction, apparent density, tensile strength, RPE retention after repeated flexing, iodine leaching stability, and X-ray shielding performance at 80 kVp. In contrast, the L5 specimen showed a tendency toward increased agglomeration and structural heterogeneity associated with excessive addition of lignin, which was accompanied by reductions in mechanical properties and shielding performance.

The deterioration in performance observed in the PU-Bi-I-L5 sample may be associated not only with self-aggregation of lignin but also with the relative reduction in the PU matrix content. In this study, the PU content decreased from 20 wt% in the L0 sample to 15 wt% in the L5 sample. This reduction in PU content may have compromised the continuity of the matrix required to adequately wet and immobilize the highly loaded Bi_2_O_3_/iohexol fillers, which could potentially result in increased filler-rich domains, microvoids, and interfacial defects. In addition, the increase in mixture viscosity with increasing lignin content may have hindered leveling and degassing during the casting process and thus adversely affected film formation. Therefore, the increased EDS distribution CV and void fraction together with the deterioration in tensile properties and shielding performance observed in L5 may be attributed to the combined effects of lignin self-aggregation and the reduced PU matrix content.

This study does involve some notable limitations. The results were obtained using prototype sheets with a thickness of 0.3 mm and therefore should not be interpreted as demonstrating an immediate replacement for commercial radiation-protective aprons. Furthermore, the interaction between lignin and the iodine-containing component was not directly demonstrated by spectroscopic analysis. Therefore, the proposed interaction should be regarded as an indirect interpretation based on the combined findings from FESEM-EDS analysis, iodine leaching stability, and retention of shielding performance. Future studies should consider further validation under broad-beam and scattered-radiation conditions, long-term iodine leaching stability under exposure to artificial sweat, simulated body fluid (SBF), disinfectants, and repeated washing, and direct characterization of lignin–iodine interactions using techniques such as Fourier-transform infrared spectroscopy (FTIR), Raman spectroscopy, and X-ray photoelectron spectroscopy (XPS). Such investigations could extend this approach to a broader range of shielding material systems. Accordingly, the lignin-assisted iodine/Bi_2_O_3_/PU composite sheet proposed in this study may serve as a practical platform for the development of environmentally conscious and lead-free radiation shielding materials.

## 5. Conclusions

In this study, we have demonstrated that a lignin-assisted interfacial stabilizer can improve the dispersion and stability of the iodine-containing component and Bi_2_O_3_ fillers in PU-based lead-free shielding sheets containing fixed loadings of 50 wt% Bi_2_O_3_ and 30 wt% iodine-containing component. The addition of 2–3 wt% lignin resulted in more uniform distributions of I and Bi, reduced microvoids and agglomerated regions, increased apparent density, improved tensile strength, and enhanced retention of shielding performance after repeated flexing.

In the evaluation of X-ray shielding performance, the specimen containing 3 wt% lignin exhibited an RPE of 77.4%, a linear attenuation coefficient of 48.52 cm^−1^, an HVL of 0.143 mm, and an MFP of 0.206 mm at 80 kVp, demonstrating superior effective attenuation performance compared with the lignin-free specimen. In addition, after 14 days of immersion, iodine leaching decreased from 3.8% in the lignin-free specimen to 1.1–1.3% in the specimens containing 2–3 wt% lignin. This indicates that lignin may also contribute to the stabilization of the iodine-containing component and the retention of shielding performance.

These findings suggest that lignin does not serve as a substitute for high-atomic-number shielding materials but can instead function as a bio-derived auxiliary material that enhances the structural homogeneity and effective shielding performance of PU shielding sheets containing high loadings of Bi_2_O_3_ and iodine. Accordingly, a lignin content of 2–3 wt% may be proposed as an optimal addition range for flexible, environmentally conscious, lead-free auxiliary shielding sheets intended for low-energy diagnostic X-rays and low-dose scattered-radiation environments.

## Figures and Tables

**Figure 1:**
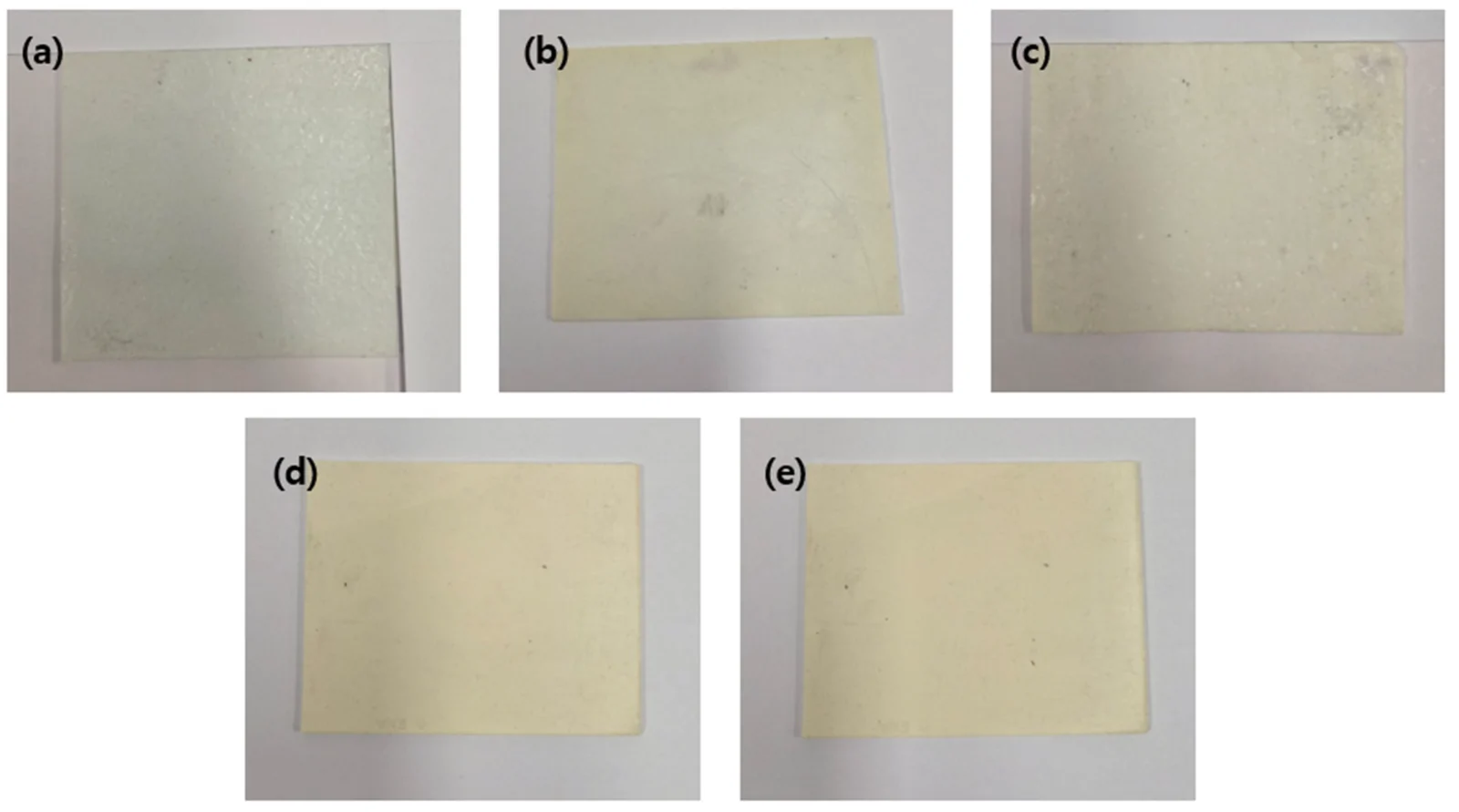
Photographs of PU/Bi_2_O_3_/iodine/lignin shielding sheets with different lignin contents. (**a**) PU-Bi-I-L0, (**b**) PU-Bi-I-L1, (**c**) PU-Bi-I-L2, (**d**) PU-Bi-I-L3, (**e**) PU-Bi-I-L5.

**Figure 2:**
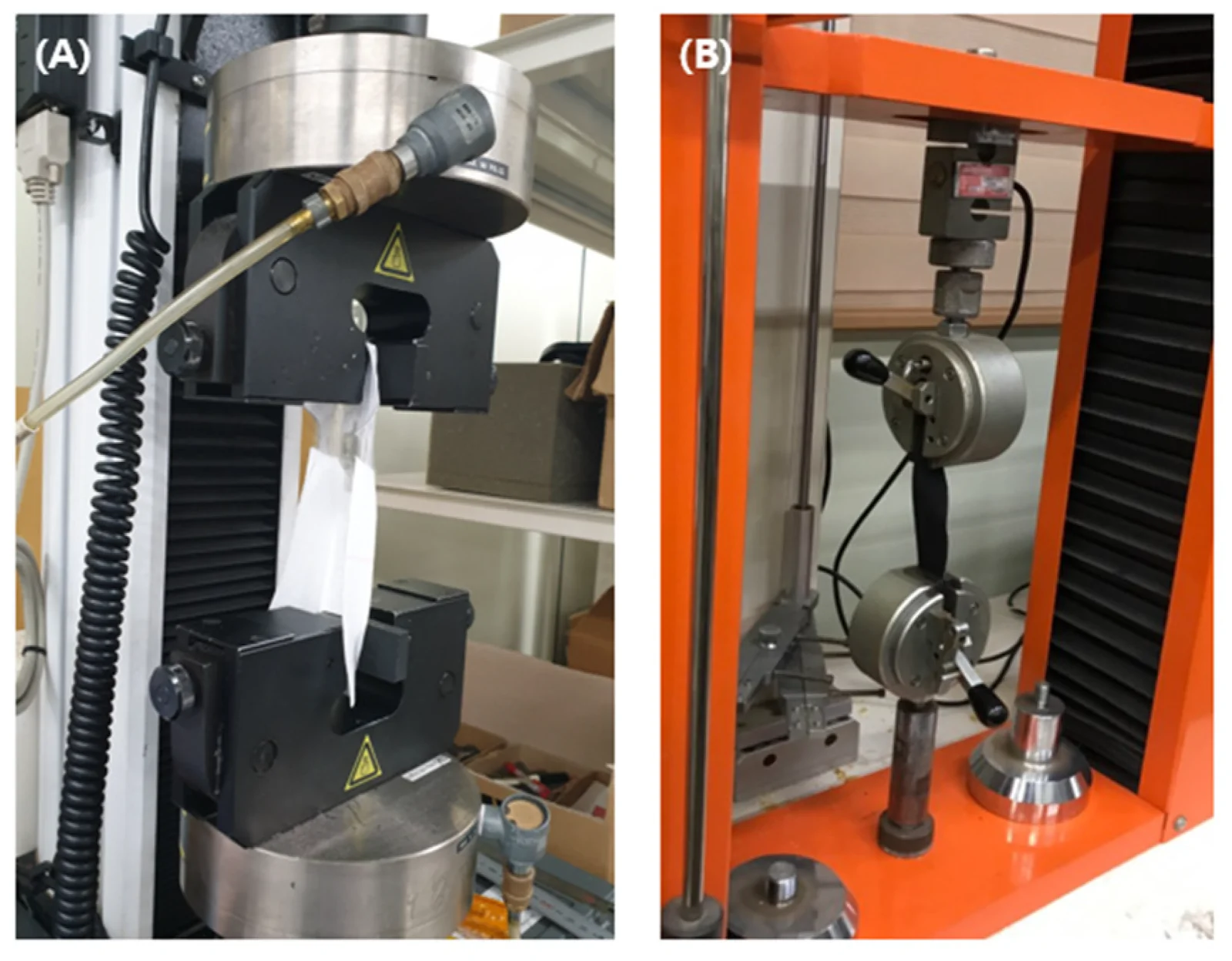
Mechanical property evaluation of the shielding sheet: (**A**) tensile testing machine and (**B**) cyclic bending test apparatus.

**Figure 3:**
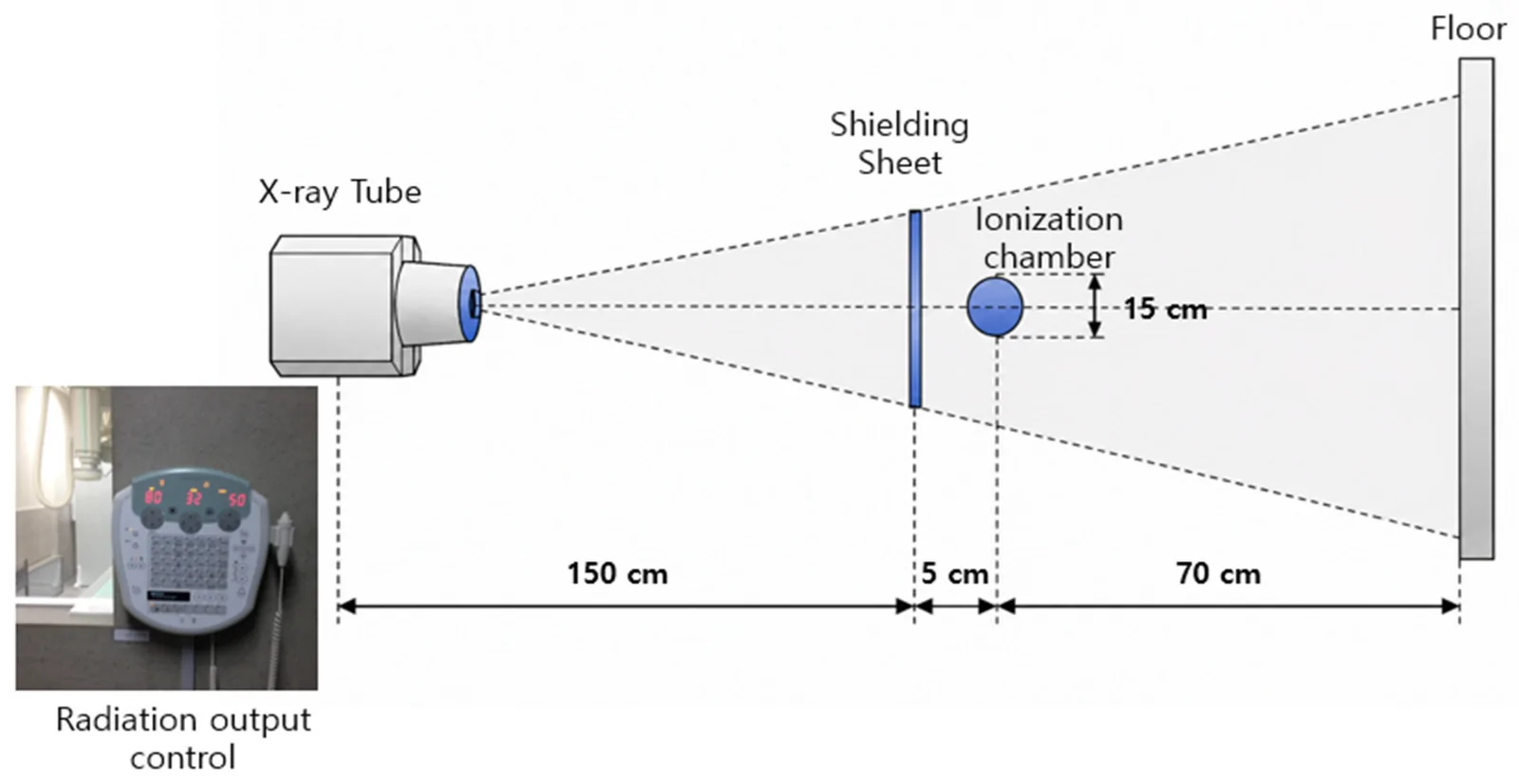
Test method used to evaluate the radiation shielding performance of the shielding sheets.

**Figure 4:**
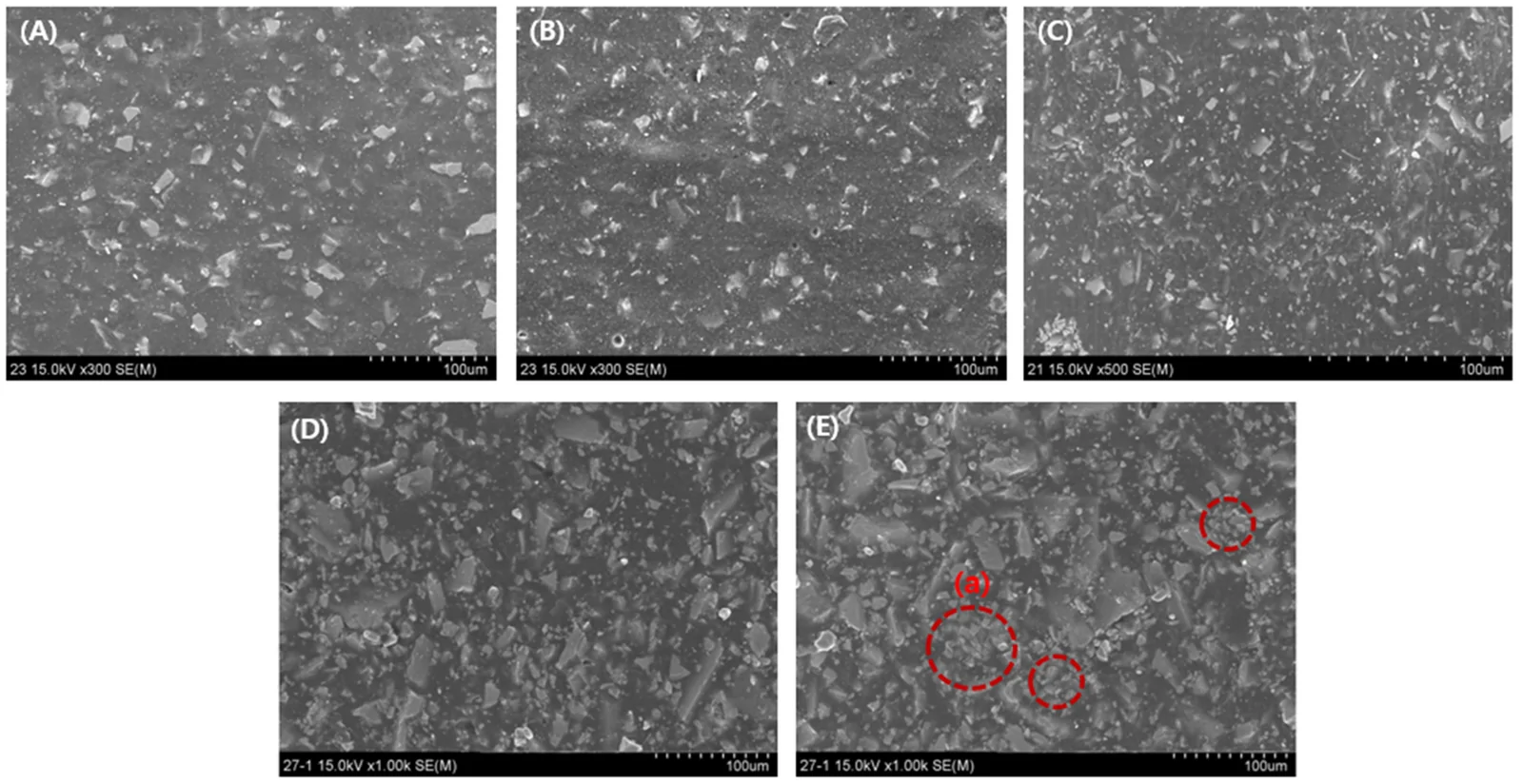
Surface morphology of the fabricated shielding sheets: (**A**) PU-Bi-I-L0, (**B**) PU-Bi-I-L1, (**C**) PU-Bi-I-L2, (**D**) PU-Bi-I-L3, and (**E**) PU-Bi-I-L5; (a) region showing fine agglomeration.

**Figure 5:**
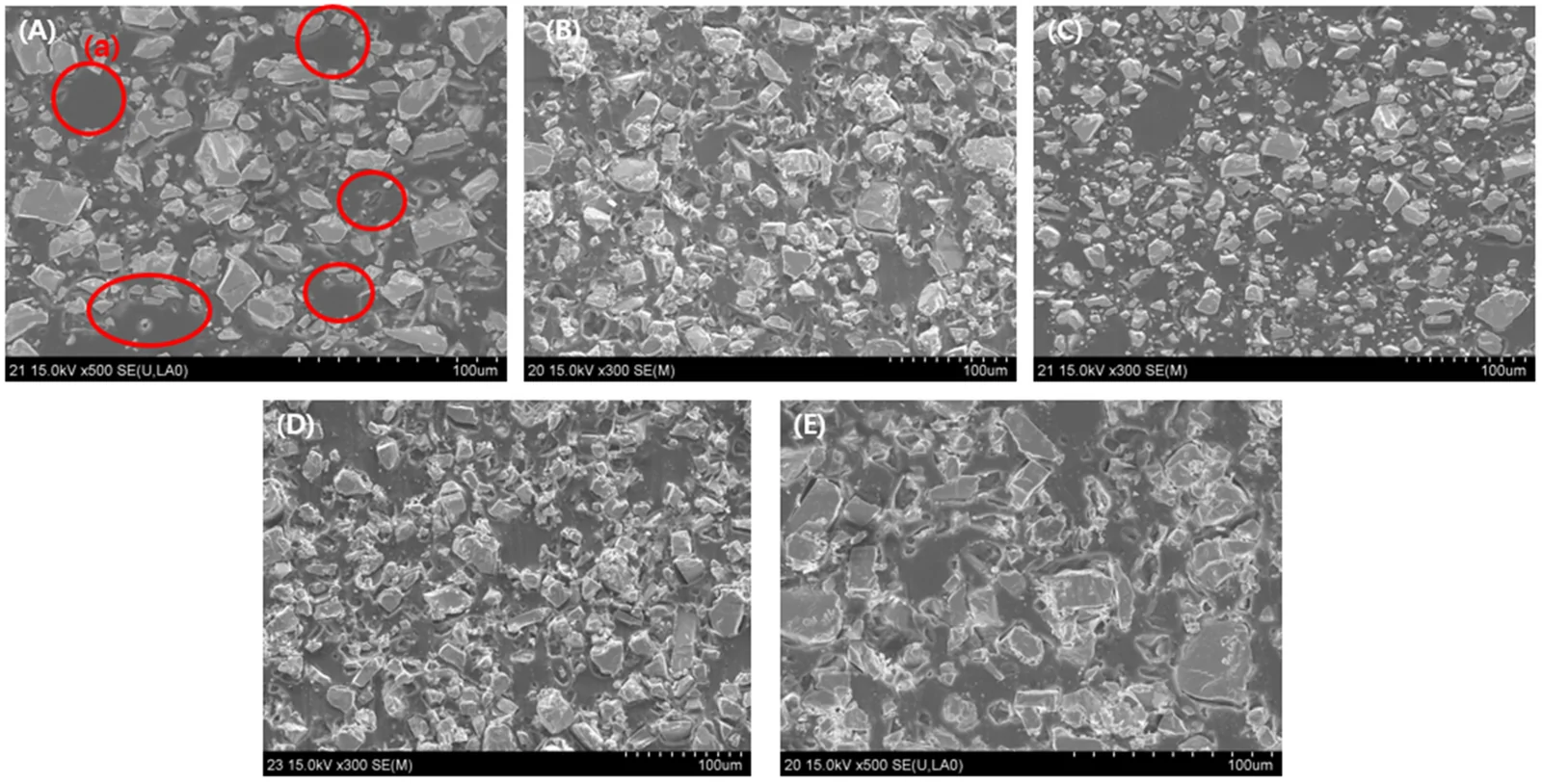
Cross-sectional image analysis of the fabricated sheets: (**A**) PU-Bi-I-L0, (**B**) PU-Bi-I-L1, (**C**) PU-Bi-I-L2, (**D**) PU-Bi-I-L3, and (**E**) PU-Bi-I-L5; (a) voids observed between particles.

**Figure 6:**
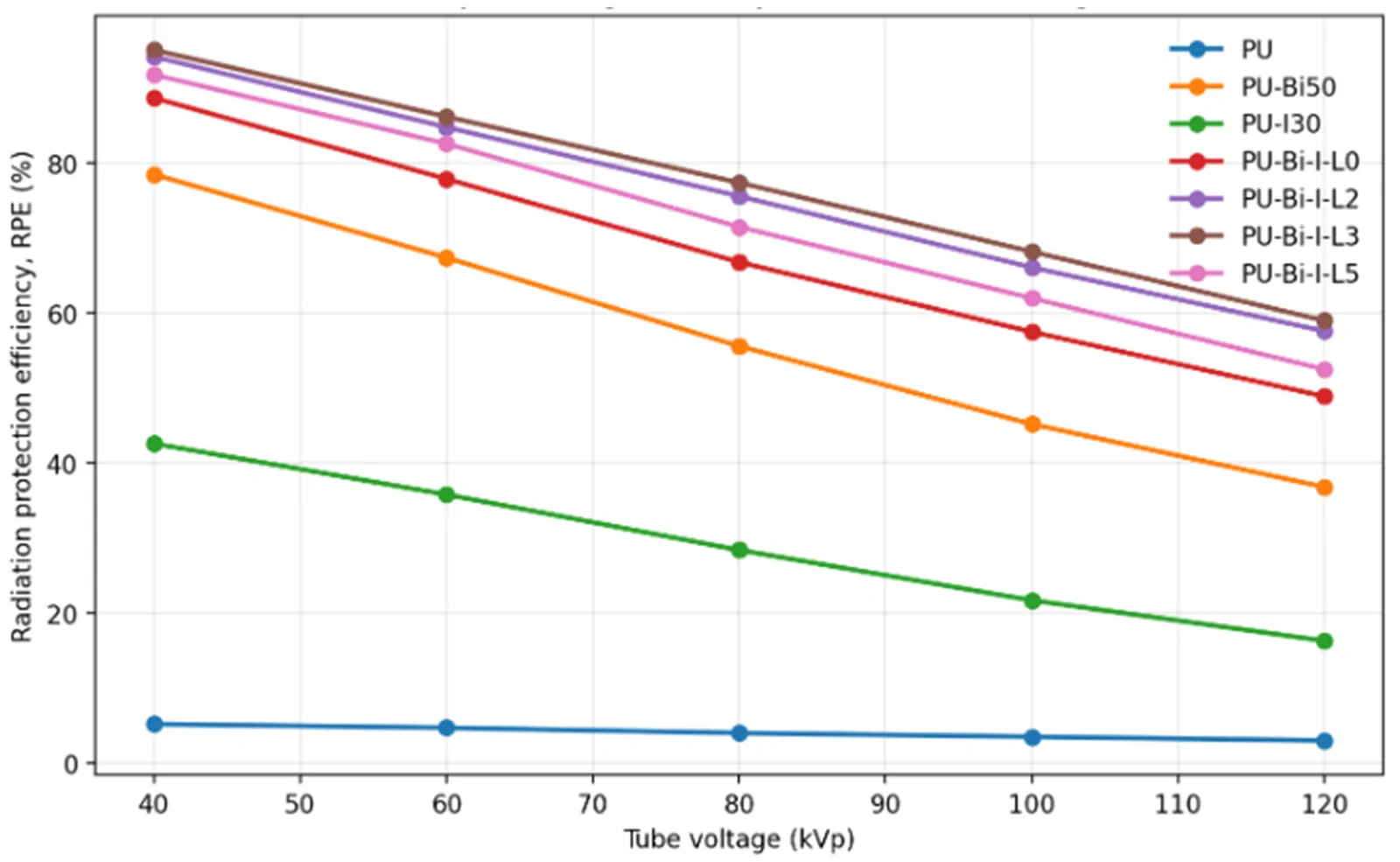
Illustrative X-ray shielding efficiency of PU/Bi2O3/iodine/iodine sheets.

**Table 1:** Composition of PU/Bi_2_O_3_/Iodine/Lignin shielding sheets.

Sample	Bi_2_O_3_ (wt%)	Iodine Component (wt%)	Lignin (wt%)	PU (wt%)	Purpose
PU-Bi50	50	0	0	50	Evaluation of the shielding performance of Bi_2_O_3_ alone
PU-I30	0	30	0	70	Evaluation of the shielding performance of the iodine component alone
PU-Bi-I-L0	50	30	0	20	Lignin-free reference group
PU-Bi-I-L1	50	30	1	19	Evaluation of the effect of low lignin content
PU-Bi-I-L2	50	30	2	18	Optimal candidate group 1
PU-Bi-I-L3	50	30	3	17	Optimal candidate group 2
PU-Bi-I-L5	50	30	5	15	Evaluation of the limitations of excessive lignin addition

**Table 2:** EDS distribution uniformity and microstructural defects according to lignin content.

Sample	Distribution CV, %		Void Area Fraction, %	Agglomerate Area Fraction, %
	I	Bi		
PU-Bi-I-L0	18.6 ± 1.9	15.4 ± 1.5	3.7 ± 0.5	6.1 ± 0.7
PU-Bi-I-L1	13.9 ± 1.4	12.2 ± 1.1	2.6 ± 0.4	4.2 ± 0.5
PU-Bi-I-L2	9.8 ± 1.0	8.7 ± 0.8	1.6 ± 1.3	2.4 ± 0.4
PU-Bi-I-L3	8.5 ± 0.9	8.3 ± 0.8	1.4 ± 0.3	2.1 ± 0.3
PU-Bi-I-L5	14.8 ± 1.6	11.9 ± 1.3	2.8 ± 0.4	4.6 ± 0.6

**Table 3:** Thickness, mass per unit area, and apparent density of the fabricated shielding sheets.

Sample	Thickness (mm)	Areal Density (g/cm^2^)	Apparent Density (g/cm^3^)
PU-Bi50	0.301 ± 0.004	0.0645 ± 0.0014	2.14 ± 0.05
PU-I30	0.298 ± 0.005	0.0428 ± 0.0012	1.44 ± 0.04
PU-Bi-I-L0	0.304 ± 0.006	0.0765 ± 0.0016	2.52 ± 0.05
PU-Bi-I-L1	0.305 ± 0.005	0.0792 ± 0.0015	2.60 ± 0.05
PU-Bi-I-L2	0.306 ± 0.004	0.0824 ± 0.0018	2.69 ± 0.06
PU-Bi-I-L3	0.307 ± 0.005	0.0837 ± 0.0017	2.73 ± 0.06
PU-Bi-I-L5	0.312 ± 0.007	0.0818 ± 0.0023	2.62 ± 0.07

**Table 4:** Tensile strength and repeated flexing test results of the shielding sheets.

Sample	Tensile Strength (MPa)	Elongation at Break (%)	Mass Loss After Flexing (%)	RPE Retention at 80 kVp, %
PU-Bi50	6.8 ± 0.5	70 ± 8	0.9 ± 0.1	92.0 ± 2.1
PU-I30	18.2 ± 1.1	120 ± 10	0.3 ± 0.1	98.8 ± 1.0
PU-Bi-I-L0	7.4 ± 0.6	45 ± 6	0.8 ± 0.1	91.4 ± 2.0
PU-Bi-I-L1	8.6 ± 0.5	55 ± 5	0.6 ± 0.1	94.7 ± 1.5
PU-Bi-I-L2	10.9 ± 0.7	68 ± 7	0.4 ± 0.1	97.3 ± 1.1
PU-Bi-I-L3	11.2 ± 0.8	65 ± 6	0.4 ± 0.1	97.8 ± 1.0
PU-Bi-I-L5	8.4 ± 0.7	41 ± 5	1.0 ± 0.2	92.1 ± 2.3

**Table 5:** Cumulative iodine leaching and retention of shielding performance after immersion.

Sample	24 h, %	72 h, %	7 d, %	14 d, %	RPE Retention After 14 d at 80 kVp, %
PU-Bi-I-L0	1.2 ± 0.2	2.0 ± 0.3	3.1 ± 0.4	3.8 ± 0.5	92.1 ± 1.8
PU-Bi-I-L2	0.4 ± 0.1	0.7 ± 0.1	1.0 ± 0.2	1.3 ± 0.2	98.0 ± 1.0
PU-Bi-I-L3	0.35 ± 0.1	0.6 ± 0.1	0.9 ± 0.2	1.1 ± 0.2	98.4 ± 0.9
PU-Bi-I-L5	0.9 ± 0.2	1.5 ± 0.3	2.1 ± 0.3	2.7 ± 0.4	94.2 ± 1.6

**Table 6:** RPE at different tube voltages.

Sample	40 kVp	60 kVp	80 kVp	100 kVp	120 kVp
PU-Bi50	78.5 ± 0.4	67.4 ± 1.2	55. ± 1.0	45.2 ± 1.1	36.8 ± 1.0
PU-I30	42.6 ± 1.4	35.8 ± 1.0	28.4 ± 0.8	21.7 ± 0.7	16.3 ± 0.6
PU-Bi-I-L0	88.7 ± 1.1	77.9 ± 1.0	66.8 ± 0.9	57.5 ± 0.8	48.9 ± 0.8
PU-Bi-I-L1	91.6 ± 1.0	81.9 ± 0.9	71.2 ± 0.8	61.4 ± 0.8	52.7 ± 0.7
PU-Bi-I-L2	94.2 ± 0.8	84.8 ±0.8	75.6 ± 0.7	66.1 ± 0.7	57.6 ± 0.7
PU-Bi-I-L3	95.1 ± 0.8	86.2 ± 0.7	77.4 ± 0.7	68.2 ± 0.6	59.0 ± 0.7
PU-Bi-I-L5	91.8 ± 1.1	84.2 ± 0.9	71.5 ± 0.9	65.0 ± 0.8	56.5 ± 0.8

**Table 7:** X-ray Attenuation Parameters at 80 kVp.

Sample	T = I/I_0_	RPE, %	μ, cm^−1^	μ/ρ, cm^2^/g	HVL, mm	MFP, mm
PU-Bi50	0.444	55.6	26.97	12.59	0.257	0.371
PU-I30	0.716	28.4	11.22	7.81	0.618	0.891
PU-Bi-I-L0	0.332	66.8	36.36	14.45	0.191	0.275
PU-Bi-I-L1	0.288	71.2	40.86	15.74	0.170	0.245
PU-Bi-I-L2	0.244	75.6	46.15	17.14	0.150	0.217
PU-Bi-I-L3	0.226	77.4	48.52	17.79	0.143	0.206
PU-Bi-I-L5	0.285	71.5	40.24	15.35	0.172	0.249

## Data Availability

Data are contained within the article.

## Abbreviations

The following abbreviations are used in this manuscript:

PU	Polyurethane
HVL	Half-Value Layer
MFP	Mean Free Path
CT	Computed Tomography
RPE	Radiation protection efficiency
WPU	Waterborne Polyurethane
FE-SEM	Field-emission scanning electron microscopy
EDS	Energy-Dispersive X-ray Spectroscopy
ROI	Region of Interest
CV	Coefficient of Variation
ICP-OES	Inductively Coupled Plasma Optical Emission Spectrometry
FTIR	Fourier-Transform Infrared Spectroscopy
XPS	X-ray Photoelectron Spectroscopy
